# Subpopulations of Circulating Cells with Morphological Features of Malignancy Are Preoperatively Detected and Have Differential Prognostic Significance in Non-Small Cell Lung Cancer

**DOI:** 10.3390/cancers13174488

**Published:** 2021-09-06

**Authors:** Emanuela Fina, Davide Federico, Pierluigi Novellis, Elisa Dieci, Simona Monterisi, Federica Cioffi, Giuseppe Mangiameli, Giovanna Finocchiaro, Marco Alloisio, Giulia Veronesi

**Affiliations:** 1 Humanitas Research Center, IRCCS Humanitas Research Hospital, Manzoni 56, 20089 Rozzano, MI, Italy; simonterisi@gmail.com; 2 Division of Pathology, IRCCS Humanitas Research Hospital, Manzoni 56, 20089 Rozzano, MI, Italy; davide.federico@humanitas.it; 3 Department of Biomedical Sciences, Humanitas University, Rita Levi Montalcini 4, 20072 Pieve Emanuele, MI, Italy; giuseppe.mangiameli@cancercenter.humanitas.it (G.M.); marco.alloisio@cancercenter.humanitas.it (M.A.); 4 Division of Thoracic Surgery, Humanitas Cancer Center, IRCCS Humanitas Research Hospital, Manzoni 56, 20089 Rozzano, MI, Italy; pierluigi.novellis84@gmail.com (P.N.); dieci.elisa@hsr.it (E.D.); federica.cioffi@humanitas.it (F.C.); 5 Division of Oncology and Hematology, IRCCS Humanitas Research Hospital, Manzoni 56, 20089 Rozzano, MI, Italy; giovanna.finocchiaro@cancercenter.humanitas.it

**Keywords:** circulating tumor cells, lung cancer, early diagnosis, cancer biomarkers

## Abstract

**Simple Summary:**

Lung cancer is by far the main cause of cancer-related deaths among both men and women. Early detection of malignant nodules and non-invasive monitoring of disease status is essential to increase the chance of cure. In this study, we analyzed the frequency and the biological features of circulating tumor cells, i.e., cells released from the tumor and in transit in the bloodstream, in patients with a diagnosis of non-small cell lung cancer undergoing surgical resection, with the aim to develop a blood-based diagnostic test and to promptly identify patients at risk of post-operative disease recurrence.

**Abstract:**

Background: Non-small cell lung cancer (NSCLC) frequently presents when surgical intervention is no longer feasible. Despite local treatment with curative intent, patients might experience disease recurrence. In this context, accurate non-invasive biomarkers are urgently needed. We report the results of a pilot study on the diagnostic and prognostic role of circulating tumor cells (CTCs) in operable NSCLC. Methods: Blood samples collected from healthy volunteers (*n* = 10), nodule-negative high-risk individuals enrolled in a screening program (*n* = 7), and NSCLC patients (*n* = 74) before surgery were analyzed (4 mL) for the presence of cells with morphological features of malignancy enriched through the ISET^®^ technology. Results: CTC detection was 60% in patients, while no target cells were found in lung cancer-free donors. We identified single CTCs (sCTC, 46%) and clusters of CTCs and leukocytes (heterotypic clusters, hetCLU, 31%). The prevalence of sCTC (sCTC/4 mL ≥ 2) or the presence of hetCLU predicted the risk of disease recurrence within the cohort of early-stage (I–II, *n* = 52) or advanced stage cases (III–IVA, *n* = 22), respectively, while other tumor-related factors did not inform prognosis. Conclusions: Cancer cell hematogenous dissemination occurs frequently in patients with NSCLC without clinical evidence of distant metastases, laying the foundation for the application of cell-based tests in screening programs. CTC subpopulations are fine prognostic classifiers whose clinical validity should be further investigated in larger studies.

## 1. Introduction

Lung cancer is the tumor with the highest fatality rate worldwide, both in males and females [1], due to its aggressiveness and biological heterogeneity, with non-small cell lung cancer (NSCLC) representing the most frequent histological subtype [2,3]. Importantly, since approximately 75% of patients present symptoms when the disease has advanced locally or disseminated at distant sites [4], lung cancer mortality is also a large consequence of late diagnoses. Therefore, early detection is a key for improving patient’s survival [5], and the US National Lung Screening Trial (NLST), the Nelson study and other non-randomized trials have actually demonstrated a significant reduction in mortality (20–30%) and morbidity upon screening programs based on a thoracic scan by low-dose computed tomography (LDCT) [6,7]. At present, uncertainties on high costs, risk of radiation exposure and false positives are obstacles to the large-scale implementation of such screening in Europe [8,9,10], and controversies exist in the management of subjects with indeterminate or premalignant nodules, which have to be monitored for a long time, and in some cases biopsied, increasing the risk of subsequent morbidities [11]. Moreover, notwithstanding the considerable advantages for patients who are diagnosed with the early-stage disease compared to those with unresectable tumors [12,13], unfortunately, 30% to 50% of cases who receive an indication for local treatment experience disease recurrence and die despite surgery with curative intent [14,15]. Lung cancer still lacks accurate biomarkers, and staging is no longer considered an accurate prognostic factor since patients with the disease at the same stage may undergo recurrence with variable incidence [16,17]. In this context, both non-invasive diagnostic tests and novel prognostic and predictive biomarkers are urgently needed to better stratify patients at risk of recurrence upon surgery and adjuvant therapies [18,19,20].

The measurement of blood biomarkers is an attractive approach to monitor cancer appearance and evolution: (i) suitable to be repeated, (ii) minimally invasive and (iii) believed to represent the systemically diffused expression of tumor heterogeneity [21,22,23]. Among all possible non-invasive biomarkers, circulating tumor cells (CTCs) are largely informative as they represent cancer cells in transit in blood, with the expected ability to re-seed the site of origin and/or to colonize distant organs [24], and which can be enumerated and characterized at DNA, RNA, protein and morphological level, thus providing access to a considerable amount of information. Importantly, hematogenous dissemination is now considered an early event in tumor progression [25], and CTCs were actually observed in the blood of patients without clinically detectable metastases in several solid tumors [26]. In lung cancer, a seminal work demonstrated that CTC analysis with the Isolation by Size of Epithelial/Tumor cells (ISET^®^) technology, which enables the vast majority of hematopoietic cells to be excluded by blood sample filtration through a porous membrane, can anticipate the detection of malignant nodules by computed tomography scan in patients with chronic obstructive pulmonary disease (COPD) who had eventually developed stage IA tumor [27], fostering the introduction of cell-based tests in diagnostic trials.

CTCs can be enriched and detected by several techniques based on the physical and biological differences between cancer cells and blood cell types; however, accurate detection is hampered by their rarity and phenotypical heterogeneity [28]. Size-based enrichment of CTCs coupled to cytomorphological analysis are unbiased with respect to the expression of protein markers and may reach a sensitivity of one cell per blood volume [29,30]. On the basis of these considerations, we have analyzed blood samples of lung cancer-free individuals and operable NSCLC patients before surgery, using the ISET^®^ technology. Previous works in early-stage NSCLCs demonstrated that CTCs can be observed by ISET^®^ in about 50% of patients and that they have prognostic significance in the preoperative setting [31,32], while in a multicentric screening trial, CTC analysis was able to identify only 26% of lung cancers detected at first LDCT scan [33].

Here, we have described atypical cells and searched for cells with morphological features of malignancy upon staining with standard cytological colorants, with the aim to assess the prognostic significance of CTC count and possible CTC subpopulations in operable NSCLC and to further explore the applicability of a CTC-based test in screening programs by comparison with a cohort of lung cancer-free individuals.

## 2. Materials and Methods

### 2.1. Study Design

We designed and carried out a prospective observational study, following the STROBE guidelines and the approval by the Ethics Committee of Humanitas Research Hospital in Rozzano (Milan, Italy). Signed informed consent was obtained from all patients. Consecutive patients with a confirmed diagnosis of NSCLC, aged 18 years or older, not pregnant, treatment naïve, without prior cancer detected within the previous 5 years or a second malignancy if there was evidence of active disease and candidate to surgical resection were enrolled from January 2017 to September 2018 and admitted in the Thoracic Surgery Division of Humanitas Cancer Center. During this phase of the study, blood samples were collected from lung cancer patients detected outside the smoker’s health multiple action (SMAC) screening program, the same day or the day before surgical intervention. Blood samples for CTC analysis were also collected from high-risk individuals enrolled in the SMAC screening program before the LDCT scan, starting from May 2019. Inclusion criteria were: heavy smokers, i.e., ≥30 packs per year, for more than 30 years, or former-smokers aged 55 years or older, who had ceased smoking within the 15 years prior to enrollment in the study, absence of symptoms of lung cancer, such as worsening of cough, hoarseness, hemoptysis and weight loss. Exclusion criteria were: previous diagnosis of lung cancer, extrapulmonary cancer history in the last 5 years (excluding in situ tumors or skin epidermoid tumor), chest CT scan performed in the last 18 months, severe lung or extrapulmonary diseases that may preclude or invalidate appropriate therapy in case of diagnosis of malignant pulmonary neoplasia. CTC analysis was performed on individuals without LDCT-detected pulmonary nodules. Blood samples from a group of healthy volunteers aged 30–50 years were analyzed as a negative control to optimize the target cell identification. The data were collected by a review of electronic medical records. The TNM staging manual of the American Joint Committee on Cancer (AJCC) 8th edition was applied. Cases considered for survival analyses include patients whose disease recurrence or death was clearly documented and attributed to NSCLC, while patients whose status was not available were excluded.

### 2.2. Blood Collection and CTC Enrichment

Samples of peripheral venous blood were drawn from patients or lung cancer-free donors using a 21G needle, collected in K_2_EDTA BD Vacutainer^®^ tubes (Becton Dickinson Italia, Milan, Italy), preserved at room temperature under gentle agitation and processed within 1.5 h using the Isolation by Size of Epithelial/Tumor cells (ISET^®^) technology (Rarecells^®^ Diagnostics, Paris, France). Briefly, 10-mL blood samples were diluted 1:10 with a proprietary Rarecells^®^ Buffer, which lysates red blood cells, and fixed with 37% formaldehyde solution (Sigma-Aldrich, St. Louis, MO, USA) at a final concentration of about 0.7% for 10 minutes and under gentle agitation. The blood was filtered through a filtration block containing a polycarbonate membrane, which hosts ten porous filter spots (8-μm-diameter cylindrical pores), each spot representing the filtration product of 1 mL of blood. The membranes were stored at 4 °C until cytological staining.

### 2.3. CTC Detection Method and Identification Criteria

Four ISET membrane spots per sample, which are the equivalent of 4 mL of blood, were stained using May–Grünwald and Giemsa colorants following these steps: incubation with a pure May–Grünwald solution (Sigma, St. Louis, MO, USA) for 5 min, then with May–Grünwald 50% diluted in distilled water for 5 min and finally with a Giemsa solution (Sigma) 10% diluted in distilled water for 20 min. Stained membranes were mounted with Organo/Limonene Mount^TM^ mounting medium (Sigma) and examined under a bright-field microscope (Olympus BX51) using a 10× objective. Areas of interest were subsequently digitalized at a 40× magnification for cytomorphological analysis. All images were analyzed by a referral cytopathologist, without knowledge of disease status and outcome, on the basis of the classical morphological criteria of malignancy, also described by Hofman and colleagues [34], and other morphological criteria to define populations of non-malignant circulating cells, as reported by Wechsler [35]. Images of atypical cells recorded during the analysis of a training set of healthy volunteers were also taken into account to exclude cells with uncertain malignancy. CTCs were defined as cells presenting with a nucleus to cytoplasm ratio >0.75 and nucleus diameter >20 μm, and with at least one of the following characteristics: nuclear border irregularities or nuclear hyperchromasia. Clusters were defined as groups of at least two CTCs or groups of at least one CTC and at least another cell type without features of malignancy, juxtaposed or in direct contact at the cell membrane level. Clusters of cells with homogenous chromatin staining and nucleus-to cytoplasm ratio <0.70, generally oval-shaped, were defined as clusters of epithelial-like cells. Large macrophage-like cells were defined as cells with a longer diameter >30 μm, with low nucleus-to-cytoplasm ratio, abundant pale basophilic cytoplasm and showing several cell shapes, such as fusiform, tadpole-like, round or oblong. Naked nuclei were defined as hyperchromatic and irregularly shaped nuclei with a longer diameter >20 μm and without apparent cytoplasm. Samples were called CTC-positive when at least one CTC was observed in a total of four filter spots, which corresponds to 4 mL of blood. All samples were considered evaluable for cytomorphological analysis according to cellularity, the prevalence of damaged cells and staining quality.

### 2.4. Spike-in Experiments

A549 ATCC^®^ CCL-185™and NCI-H460 [H460] ATCC ^®^ HTB-177™ lung cancer cell lines were kindly provided by the European Institute of Oncology in Milan. Cells were propagated according to the instructions provided by the American Type Culture Collection (Manassas, VA, USA). Before performing spike-in experiments, the cells were detached with Trypsin-Versene^®^ solution (Lonza, Basel, Switzerland), counted using the Trypan Blue 0.4% solution as a vital dye exclusion assay (viability >99% in all tests) and injected in 10 mL of blood collected from healthy volunteers at a dilution of 1000 cells per milliliter of blood (i.e., 1000 cells per membrane spot). The spiked-in samples were incubated for 30 min at room temperature under gentle agitation until chemical fixation and filtration as described before. The membranes were stained with Hematoxylin (Histo-line Laboratories Srl, Milan, Italy) for 3 min and Eosin Y aqueous solution (Histo-line Laboratories Srl) for 1 min, or with May–Grünwald and Giemsa solutions (Sigma) as described before and observed at the Olympus BX51 under a 20× magnification objective.

### 2.5. Statistical Analysis

Associations between categorical variables were tested by Fisher’s exact test using contingency tables. Differences in discrete variables were tested using the Mann–Whitney and Kruskal–Wallis test. Linear regression analysis and Pearson’s correlation coefficient *r* were used to estimate the correlation between cell numbers and age. Cox proportional-hazards regression was used to investigate the prognostic role of CTC status or number on recurrence-free survival, with relative hazard ratios (HR) and 95% confidence intervals (CI). Significance in the probability of time-to-event was tested by log-rank tests. Each selected factor was investigated in univariable analysis. All tests for the comparison of experimental groups were performed using GraphPad Prism, version 7.04. Survival and Cox regression analyses were performed, and Kaplan–Meier plots were constructed using MedCalc, version 12.7, and SPSS, version 26.0, respectively. All tests were two-sided, and significance statements refer to a *p*-value < 0.05.

## 3. Results

### 3.1. Cancer Cell Hematogenous Dissemination Is a Frequent Event in NSCLC Patients without Clinical Evidence of Metastasis

We performed the prospective collection of blood samples before surgical intervention (*n* = 74), and we analyzed blood samples of young volunteers (*n* = 10) and high-risk lung cancer-free individuals (*n* = 7), as assessed by LDCT as negative controls (Table 1). The number of cases locally treated with curative intent was 32, 20 and 17 for stages I, II and III, respectively. Five patients had stage IV disease at baseline: three underwent only diagnostic surgical procedure at pleural or lung nodules, while two underwent surgery with radical intent, one case presenting with a big, excavated lung lesion with the paraneoplastic syndrome and no possibility to undergo chemotherapy, the other case underwent segmental resection plus resection of a local pleural lesion with partial decortication for intraoperative diagnosis of a small pleural metastasis. In the control cases, cells with features of malignancy were not observed, while some nuclei larger than 20 μm, hyperchromatic or with heterogeneous chromatin stain, without apparent cytoplasm, sometimes overlapping, were found in 7 out of the 17 (41%) cases (Supplementary Figure S1). Images were digitalized and taken into account during the morphological analysis of patients’ blood samples in order to exclude indeterminate atypical figures and avoid misleading interpretations. Of the total population of NSCLC patients, 44 cases (59.5%) were called CTC positive, as they had at least one cell with clear features of malignancy detected in 4 mL of blood, 57.5% with stage I–III and 80% with stage IVA tumor (*p*-value = 0.4922). The CTC status did not correlate with patient demographics nor with smoking habits, as also with pathologic tumor stage, histology and invasiveness features, except for a statistically significant higher CTC positivity rate observed in cases without peritumoral neoplastic angioinvasion. Interestingly, the proportion of CTC-positive cases with stage I or II NSCLC was considerable (57.7%) with respect to the limited amount of blood analyzed in this study (Table 1).

### 3.2. Subpopulations of Circulating Atypical Cells Differentiate Operable NSCLC Patients from Lung Cancer-Free Individuals

**Figure 1:**
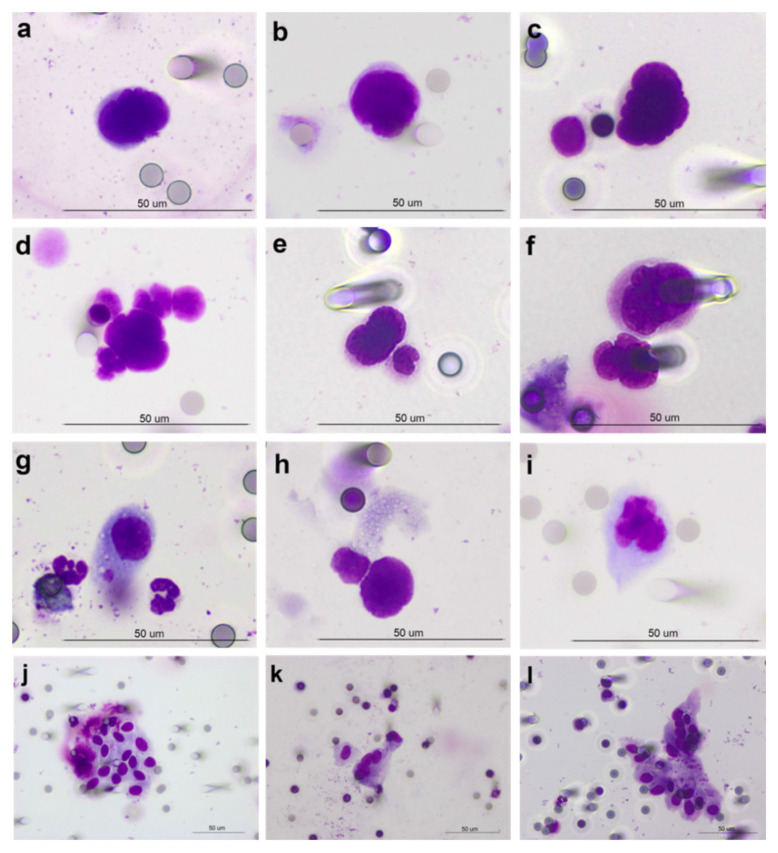
Subpopulations of atypical circulating cells differentiate patients with operable non-small cell lung cancer from lung cancer-free individuals. Images depict (**a**–**c**) single cells with morphological features of malignancy and (**d**–**f**) heterotypic clusters of cells with features of malignancy physically interacting with normal cells, such as (**d**) neutrophil granulocytes, (**e**) monocytes or (**f**) other indeterminate leukocytes, detected in patients, (**g**–**i**) atypical macrophage-like cells, (**g**) oblong or (**h**) tadpole-like, both detected in patients, and irregularly shaped (**i**), detected in healthy donors, and (**j**–**l**) clusters of epithelial-like cells, detected in patients, on porous membranes stained with May–Grünwald and Giemsa. Objective magnification 40×.

The morphological analysis of cytological samples prepared on ISET membranes revealed the presence of a heterogeneous population of circulating atypical cells, which includes different subsets observed at a variable frequency within the two cohorts of patients and controls. We identified three subpopulations of cells with features of malignancy, hereafter referred to as (i) single CTCs (*n* = 71 cells), i.e., not in direct contact with other cells (sCTC, Figure 1a–c), (ii) homotypic CTC clusters (homCLU, *n* = 2 clusters), i.e., groups of slightly overlapping CTCs, and (iii) heterotypic clusters (hetCLU, *n* = 40 clusters), i.e., CTCs in direct contact with leukocytes (Figure 1d–f), mainly monocytes (62.5%) and neutrophil granulocytes (12.5%); we also observed (iv) a subpopulation of large cells, hereafter referred to as atypical macrophage-like cells (Figure 1g–i), (v) a subpopulation of clusters of epithelial-like cells (Figure 1j–l) and (vi) a considerable number of nuclei with a longer diameter >20 μm, in some cases with irregular membrane border and/or hyperchromasia, each of them apparently without the classical cytoplasmic rim observed in CTCs, in some cases similar to those observed in healthy donors (Supplementary Figure S1), hereafter referred to as naked nuclei (Supplementary Figure S2). In order to exclude the possibility that clusters were a result of technical artifacts due to the CTC enrichment procedure, we performed spike-in experiments with A549 and NCI-H460 cell lines at a dilution of about 1000 cells per milliliter in a total of 10 mL of blood from three healthy donors. Spiked-in, filtered and stained tumor cells were typically round-shaped and showed nucleus diameters larger than 20 μm, a nucleus-to-cytoplasm ratio around 90% and nuclear hyperchromatism. We did not observe the formation of homotypic clusters after filtration and staining, except for some doublets of NCI-H460 cells, and leukocytes were randomly interspersed among cancer cells.

**Figure 2:**
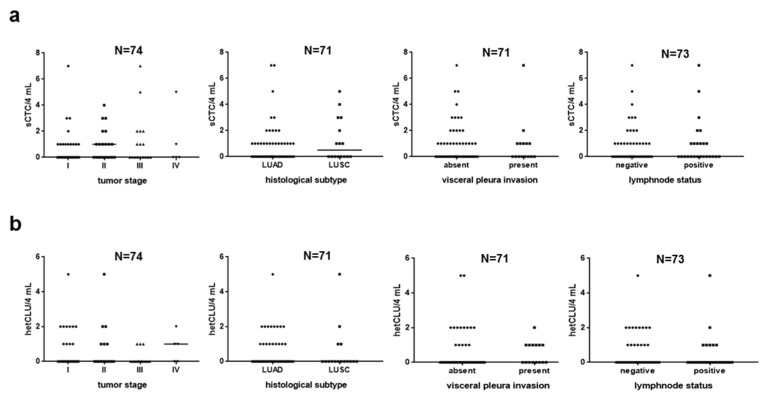
The number of single CTCs (sCTC) and heterotypic CTC clusters (hetCLU) is not associated with the clinicopathological features of stage I–IVA non-small cell lung cancers. Dot plots represent the distribution of (**a**) sCTC or (**b**) hetCLU count (median number, line) in 4 mL of peripheral blood according to the tumor stage, the histological subtype (LUAD, lung adenocarcinoma; LUSC, lung squamous cell carcinoma), the visceral pleura invasion and the lymph-node involvement. Differences were not significant (*p*-value > 0.05) by Kruskal–Wallis (tumor stage) or Mann–Whitney test.

In the cohort of 74 NSCLC patients, sCTC and hetCLU were detected in 34 (45.9%) and 23 (31.1%) cases, respectively, while both CTC subsets were observed in 14 (18.9%) cases. Homotypic clusters were observed in two cases only (2.7%). Neither the presence nor the prevalence or number of sCTC correlated with patients’ demographics, smoking habits and tumor features, except for males and smokers, where at least two sCTC were detected at a significantly higher frequency compared to females and never smokers (Supplementary Table S1 and Figure 2a). Neither the presence nor the number of hetCLU correlated with the clinicopathological features (Supplementary Table S2 and Figure 2b).

Cells with features of atypical large macrophages were found in 12 out of 74 (16.2%) patients, and 2 out of 17 (12%) controls, whereas naked nuclei were observed in both cohorts, although with a two-fold increased frequency in patients (61/74, 82.4%) compared to controls (7/17, 41%; *p*-value = 0.0011). Interestingly, clusters of epithelial-like cells without apparent features of malignancy were detected in 9 out of 74 patients (12.2%) and none of the control cases. The number of patients called CTC-positive, who also had at least one atypical large macrophage, naked nucleus or cluster of epithelial-like cells were 11 (25%), 37 (84.1%) and 4 (9.1%), respectively.

### 3.3. The Prevalence of Single Circulating Tumor 00000Cells Predicts the Risk of Recurrence in Patients with Surgically Treated Stage I–II NSCLCs

**Figure 3:**
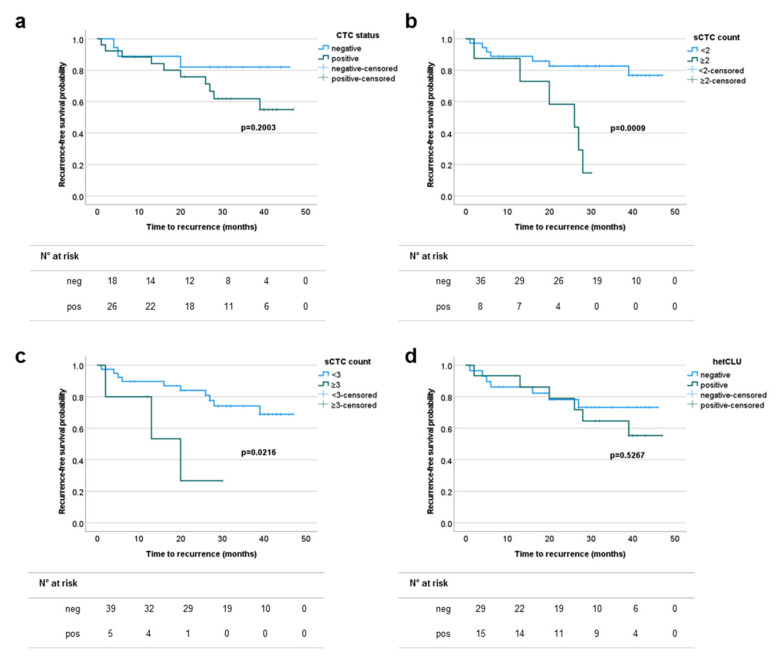
The prevalence of single circulating tumor cells (sCTC) correlates with reduced recurrence-free survival in stages I and II non-small cell lung cancers. Kaplan–Meier plots showing the time-to-event probability of disease recurrence according to (**a**) the presence of CTCs irrespective of the cell subset, (**b**,**c**) the prevalence of sCTC or (**d**) the presence of heterotypic circulating tumor cell clusters (hetCLU).

We then explored the utility of CTCs in serving as prognostic biomarkers to identify patients with an early-stage tumor at higher risk of disease recurrence. All stage I and II cases underwent surgical resection with curative intent. Six out of the 52 patients received post-operative adjuvant platinum-based with either vinorelbine or gemcitabine chemotherapy and/or radiotherapy. The disease status of all patients was monitored by clinical and radiological exams, except for eight cases (two stage I and six stage II) who were lost at follow-up (*n* = 3) or had died for unknown causes (*n* = 5). The median (range) observation time was 28 (1–47) months, and the total number of recurrence events was 13 (seven at intrathoracic level, five at distant sites and missing information in one case). The risk of disease recurrence in stages III and IV compared to stages I and II patients were HR 95%CI 3.45 (1.37–8.67), *p*-value = 0.0006, with an equal number of events (13) per group. In the stages I and II cohort, neither the tumor stage nor the grading or the lymph-node status were able to discriminate patients at higher risk of early disease recurrence. The numbers of stages I and II cases out of 44 evaluable for disease recurrence and with at least one, two, three or five sCTC, or at least one hetCLU, or CTC-positive irrespective of the subset type, were 21 (47.7%), 8 (18.2%), 5 (11.4%), 1 (2.3%), 15 (34.1%) and 26 (59.1%), respectively. No differences were found when grouping patients according to the overall CTC status or the presence of hetCLU, while patients with a prevalence of at least two or three CTCs in 4 mL of blood had a statistically significant shorter recurrence-free survival probability (HR 95% CI, cut-off two CTC/4 mL: 5.15 (1.10–24.33), *p*-value = 0.0009; cut-off three CTC/4 mL: 3.99 (0.47–33.57), *p*-value = 0.0216) compared to the counterpart with a more favorable CTC count (Figure 3 and Supplementary Table S3), demonstrating that the number of sCTC was the most relevant predictor of prognosis in early-stage operable NSCLCs.

### 3.4. The Presence of Heterotypic Clusters of CTCs Predicts the Risk of Recurrence in Patients with Surgically Treated Stage III–IVA NSCLCs

We assessed the clinical significance of CTCs in the group of patients presenting with operable NSCLC at advanced stages (III and IV). Seventeen out of the 22 patients received post-operative adjuvant platinum-based with either vinorelbine or gemcitabine chemotherapy and/or radiotherapy or targeted therapy with gefitinib or afatinib for stage IV EGFR mutated tumors. The disease outcome of all patients was monitored by clinical and radiological exams, except for four stage III cases that were lost at follow-up (*n* = 1) or had died for unknown causes (*n* = 3). The median (range) observation time was 17 (1–33) months, and the total number of recurrence events was 13 (5 at intrathoracic level, 6 at distant sites and missing information in 2 cases).

**Figure 4:**
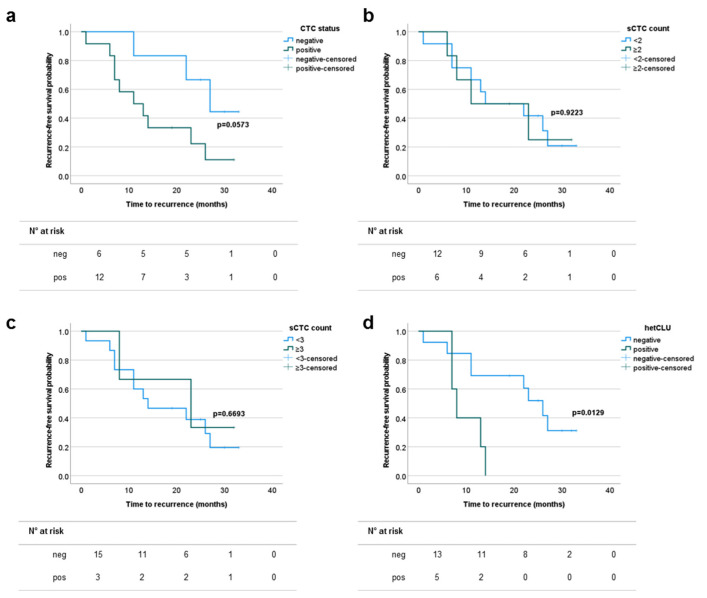
The presence of heterotypic circulating tumor cell clusters (hetCLU) correlates with reduced recurrence-free survival in stages III and IVA non-small cell lung cancers. Kaplan–Meier plots showing the time-to-event probability of disease recurrence according to (**a**) the presence of CTCs irrespective of the cell subset, (**b**,**c**) the prevalence of single CTCs (sCTC) or (**d**) the presence of hetCLU.

In advanced NSCLC patients, neither the tumor stage nor the grading were able to accurately identify cases at higher risk of early disease recurrence, although a slight trend toward statistical significance was obtained when grouping according to the tumor stage (Supplementary Table S4). The numbers of stages III and IV cases out of 18 evaluable for disease recurrence and with at least one, two, three or five sCTCs, or at least one hetCLU, or CTC-positive irrespective of the subset type, were 8 (44.4%), 6 (33.3%), 3 (16.7%), 3 (16.7%), 5 (27.8%) and 12 (66.7%), respectively. According to the survival analysis based on the CTC status, a slight trend toward statistical significance was observed when considering the overall CTC population, while no difference was found when grouping patients according to the prevalence of sCTC. Interestingly, cases presenting with at least one hetCLU in 4 mL of blood had a statistically significant shorter recurrence-free survival probability (HR 95%CI: 3.44 (0.76–15.50), *p*-value = 0.0129) compared to patients without hetCLU (Figure 4 and Supplementary Table S4), providing evidence for the first time of the prognostic significance of hetCLU in advanced stage operable NSCLCs.

## 4. Discussion

In this study, we have observed that cells with morphological features of malignancy can be detected in 4 mL of peripheral blood in 60% of patients with operable NSCLC and that the CTC frequency is not dependent on the tumor stage. We have found that in NSCLC, cells with features of malignancy can circulate in physical contact with leukocytes, mainly monocytes, forming aggregates of two cells in the majority of cases, here called heterotypic clusters, with an overall frequency of 31%, without significant differences according to the disease stage. We have also documented the presence and frequency of other subpopulations of cells in patients, such as clusters of epithelial-like cells, large macrophage-like cells and naked nuclei >20 μm, which were found both in patients and lung cancer-free controls. Importantly, our study revealed different clinical messages hidden in CTCs based on the subset type as, compared to other tumor-related factors, only the CTC number at baseline was able to inform early-stage patients’ risk and time of recurrence, whereas heterotypic clusters represented the most informative subpopulation of CTCs with respect to disease outcome in the advanced stage setting.

Contrarily to the traditional view that cancer cells disseminate via blood vessels within a time window closer to the clinical manifestation of secondary lesions rather than to the initiation of a tumor, CTCs are now widely recognized as events that can be detected at a considerable frequency even in patients presenting with early-stage or locally advanced disease. Since CTCs are known to be heterogeneous within the same patient, and a gold standard approach for accurate detection has not been developed yet [36], it is clear that the operational definition for the measurement of CTCs may change from study to study along with the technical approach. One of the first reports on CTC analysis in stage I–III NSCLC demonstrated that the CTC detection rate obtained by the capture of cells expressing EpCAM and cytokeratins 8/18/19 was lower compared to parallel samples analyzed by a size-based isolation approach coupled to cytomorphological analysis [32]. However, two main biological and technical aspects should be taken into account: first, some CTCs might have shaped their makeup while undergoing the epithelial-to-mesenchymal transition (EMT), thus downregulating the expression of surface adhesion proteins [37] and remaining undetectable to EpCAM-based capture; second, neutrophils have a low-to-absent expression of CD45 and, importantly, may be non-specifically bound by antibodies for some intracellular proteins, including cytokeratins [38], thus leading to CTC misidentification. Following the first demonstration that the early detection of CTCs was able to anticipate stage I lung cancer diagnosis by radiological scan in COPD patients [27], the majority of successive studies was performed using CTC enrichment methods based on the physical properties of tumor cells. The CTC positivity data obtained in our study population was 59.5%, which is slightly higher compared to the study by Hofman and colleagues in a case series of 210 NSCLC patients undergoing radical surgery [32], although the difference is not negligible if considering that we have analyzed 4 mL rather than 10 mL of blood. In other studies, the CTC detection rate was 80% by morphological analysis on 10 mL of blood from 40 chemonaïve stage IIIA and IV cases [39] and 69.5% by immunostaining for EpCAM and CD45 upon peripheral blood mononuclear cell collection and subsequent filtration in 82 cases with any stage [40].

In addition to the population of single CTCs, other subsets of cells are emerging as possible diagnostic biomarkers, as they showed high specificity in distinguishing between patients from lung cancer-free individuals: CTC clusters [41,42,43], which are aggregates of at least two CTCs in physical contact, held together through intercellular junctions [44], also known as circulating tumor microemboli, which can appear with infiltrated or surrounding platelets [35], or aggregates of cancer cells with non-malignant stromal or immune cells [44], as also large macrophage-like cells [35] and tumor-macrophage hybrid cells [45,46], which instead express both epithelial and leukocyte/macrophage-specific markers. The frequency of CTC clusters may vary depending on the technical approach and the disease stage [41,42,43,47]; therefore, further studies are needed to confirm the origin of this subset of CTCs. However, it has been becoming increasingly clear that CTC clusters may act as predictors or players in therapy resistance [48,49], and experimental evidence showed that polymorphonuclear/myeloid-derived suppressor cells interact with CTCs and promote their metastatic potential [50]. Remarkably, a non-conventional approach for CTC isolation recently showed that heterotypic clusters could be detected at a relevant frequency in many non-metastatic and metastatic solid tumors [51]. Therefore, heterotypic clusters can represent a promising biomarker and therapeutic target. Furthermore, the role of clusters of epithelial-like cells that we have observed in our cohort of patients is worth being clarified.

Studies with other technical approaches provided interesting results on the clinical role of CTCs in NSCLC in the operative setting. It was reported that cytokeratin- and EGFR-positive cells enriched by an immunomagnetic approach could be observed in stage I–III NSCLC patients at different frequencies before and 1 month after surgery and that post- but not pre-surgery detected cytokeratin-positive CTCs were associated with disease-free survival [52]. In 2019, a work with the CellSearch^TM^ system showed that tumor cells collected from the pulmonary vein during surgical procedures could be observed in 48% of cases, that using a cut-off of at least 18 CTCs in 7.5 mL of blood it was possible to predict disease relapse and that mutations in CTCs largely overlapped with those found in metastases detected 10 months later [53]. Other authors challenged the effect of surgery on CTC kinetics and found a significant decline in EpCAM-enriched CTCs a few days after surgery in all patients, and that early rebound of CTC counts was associated with disease recurrence [54]. Our data provide further evidence of the role of CTCs detected in the peripheral venous blood, and importantly, of the different significance that CTC subsets may have in classifying patients at risk of relapse when detected before surgery. Longitudinal studies are of interest to assess CTC kinetics in response to post-surgery administered systemic therapies.

Size-based approaches have already been shown to increase the sensitivity in detecting CTC clusters in metastatic NSCLCs compared to epitope-dependent methods. In 2011, vimentin-positive and cytokeratin-negative CTC clusters were described in three out of six metastatic NSCLC patients [55], and later it was found that circulating tumor microemboli, which were defined as clusters of at least three CTCs, could be observed in 43% of patients using ISET^®^ but were undetectable by CellSearch^TM^, which captures cells by anti-EpCAM antibodies [39]. Moreover, CTC clusters isolated by a biomarker-independent, size-based microfluidic method could be observed in 96% of patients with metastatic NSCLC, and 75% of them were EpCAM-negative [47]. In our study, with stage I–IVA NSCLC patients, the frequency of homotypic CTC clusters was negligible. The marker-free technical approach based on the direct evaluation of cytological samples enabled the visualization of CTCs in contact with leukocytes, as also of other atypical cells. The application of a filtration method coupled to morphological analysis brings many advantages. However, a crucial point that should be addressed is the reproducibility of the analysis based on cytomorphological criteria. Although they have been already defined and blindly validated by a team of 10 cytopathologists on 808 blood samples analyzed by the ISET method, such criteria hold the same limits as those used in routine cytology [56]. In our study, cells with features of malignancy were not found in lung cancer-free individuals, following blinded analysis. Larger cohorts of individuals at risk of lung cancer and the inter-reader variability should be evaluated in multicentric studies in order to increase the robustness of the CTC test and to foster its application in the clinical routine. The identification of a panel of biomarkers for CTC detection in NSCLC is also of crucial importance in this context. Searching for lung cancer-specific markers is a long-standing issue for pathologists, which consequently affects CTC studies. An attentive look at CTC and metastasis biological hallmarks may help to identify markers alternative to cytokeratin, and in fact, a recent paper showed that the glycolysis enzyme hexokinase 2 (HK2) increased the detection of CTCs in a cohort of 18 stage III lung adenocarcinoma patients without clinical evidence of distant metastases from 39% when considering cytokeratin-positive to 61% when considering HK2-positive cell subsets [57].

To summarize the results of this study, we have discovered a population of heterotypic CTC clusters in early-stage NSCLC and provided first evidence of the differential prognostic significance of single CTC count, using a low cut-off (two CTCs in 4 mL of blood), and of the presence of heterotypic clusters, in operable NSCLC patients with stages I or II and stages III or IVA, respectively, demonstrating that looking at CTC subsets rather than the overall CTC population can help to refine the classification of patients at risk of disease recurrence, and outperforming classical primary tumor-related markers; we have performed the analysis on cytological samples corresponding to 4 mL only compared to larger blood volumes (from 7.5 to 10 mL) used in other studies, scoring as CTC-positive about 60% of patients and none of the control individuals, and we have documented and described other subsets of circulating atypical cells occurring with different frequency in patients and lung cancer-free donors.

## 5. Conclusions

The ISET^®^ technology for CTC enrichment coupled to cytomorphological analysis was proven as a promising approach for the development of non-invasive biomarkers in NSCLC. With a view to the implementation of a CTC-based test in screening programs, studies with larger case series and the introduction of molecular analyses to infer the origin of atypical cells would be desirable. CTC subsets also deserve consideration to be included in the clinical routine among the standard prognostic factors in order to early identify patients at risk of recurrence and refine the therapeutic strategies accordingly.

## Figures and Tables

**Table 1:** Circulating tumor cell (CTC) detection rate and cohorts’ characteristics.

	N (%)	N CTC+ve (%)	*p*-Value
**Patients with NSCLC**	74 (100)	44 (59.5)	
Median (range) Age (years)	71 (43–86)		
**Sex**			
Female	33 (44.6)	18 (54.5)	
Male	41 (55.4)	26 (63.4)	0.4820
**Smoking habits**			
Current smoker	25 (33.8)	17 (68.0)	
Former smoker	32 (43.2)	18 (56.3)	
Never smoker	16 (21.6)	9 (56.3)	
Missing	1 (1.4)	0	0.7764 ^a^
**Tumor stage**			
IA	27 (36.5)	15 (55.6)	
IB	5 (6.8)	2 (40.0)	
IIA	4 (5.4)	4 (100)	
IIB	16 (21.6)	9 (56.3)	
IIIA	11 (14.9)	7 (63.6)	
IIIB	6 (8.1)	3 (50.0)	
IIIC	0	0	
IVA	5 (6.8)	4 (80.0)	0.7964 ^b^
**Histology**			
Adenocarcinoma	55 (74.3)	33 (60.0)	
Squamous cell carcinoma	16 (21.6)	10 (62.5)	
Other	2 (2.7)	1 (50.0)	
Missing	1 (1.4)	0	>0.9999 ^c^
**Grading**			
G1	4 (5.4)	3 (75.0)	
G2	44 (59.5)	23 (52.3)	
G3	24 (32.4)	17 (70.8)	
G4	0	0	
Missing	2 (2.7)	1 (50.0)	0.2092 ^d^
**Visceral pleura invasion**			
PL0	56 (75.7)	31 (55.4)	
PL1	8 (10.8)	6 (75.0)	
PL2	5 (6.8)	3 (60.0)	
PL3	2 (2.7)	2 (100)	
Missing	3 (4.1)	2 (66.7)	0.2494 ^e^
**Peritumoral neoplastic angioinvasion**			
Absent	66 (89.2)	42 (63.6)	
Present	6 (8.1)	1 (16.7)	
Missing	2 (2.7)	1 (50.0)	0.0357
	N (%)	N CTC + ve (%)	p-value
**Lymph-node status**			
Negative	48 (64.9)	29 (60.4)	
Positive	25 (33.8)	14 (56.0)	
Missing	1 (1.4)	0	0.8039
**Healthy volunteers**	10		
Median (range) Age (years)	35 (33–47)		
**Sex**			
Female	7 (70.0)	0	-
Male	3 (30.0)	0	-
**Smoking habits**			
Smoker	2 (20.0)	0	-
Never smoker	8 (80.0)	0	-
**High-risk subjects**	7		
Median (range) Age (years)	63 (53–73)		
**Sex**			
Female	3 (42.9)	0	-
Male	4 (57.1)	0	-

^a^ current/former versus never smokers. ^b^ stage I/II versus III/IV. ^c^ adenocarcinoma versus squamous cell carcinoma. ^d^ G1/G2 versus G3/G4. ^e^ PL0 vs. PL1/PL2/PL3.

## Data Availability

Data is contained within the article or supplementary material.

## Supplementary Materials

The following are available online at https://www.mdpi.com/article/10.3390/cancers13174488/s1, Figure S1. Naked nuclei detected in the blood of lung cancer-free individuals; Figure S2. Naked nuclei detected in the blood of on-small cell lung cancer patients; Table S1. Single circulating tumor cell (sCTC) prevalence and non-small cell lung cancer (NSCLC) patients’ characteristics; Table S2. Circulating tumor cell cluster (cCTC) status and NSCLC patients’ characteristics; Table S3. Recurrence-free survival probability in patients with operable stage I–II NSCLC according to standard clinico--pathological parameters and CTC status; Table S4. Recurrence-free survival probability in patients with operable stage III–IV NSCLC according to standard clinico-pathological parameters and CTC status.
