# Supplementary material for: Subpopulations of Circulating Cells with Morphological Features of Malignancy Are Preoperatively Detected and Have Differential Prognostic Significance in Non-Small Cell Lung Cancer
Source: Cancers (Basel). 2021 Sep 6;13(17):4488. doi: 10.3390/cancers13174488 (PMC8431290; doi:10.3390/cancers13174488)
Supplement: Supplementary file 1 [file cancers-13-04488-s001.zip › cancers-1343832-supplementary.pdf]

ONLINE SUPPLEMENT

Subpopulations Of Circulating Cells With Morphological Features Of Malignancy Are Preoperatively Detected And Have Differential Prognostic Significance In Non-Small Cell Lung Cancer

Emanuela Fina, Davide Federico, Pierluigi Novellis, Elisa Dieci, Simona Monterisi, Federica Cioffi, Giuseppe Mangiameli, Giovanna Finocchiaro, Marco Alloisio, Giulia Veronesi

Index

Supplementary Tables ………………………………………………………………………….. **2**

Supplementary Table 1. Single circulating tumor cell (sCTC) prevalence and non-small cell lung cancer (NSCLC) patients' characteristics …………………...…. 2

Supplementary Table 2. Circulating tumor cell cluster (cCTC) status and NSCLC patients' characteristics ……………………………………………………..... 4

Supplementary Table 3. Recurrence-free survival probability in patients with operable stage I-II NSCLC according to standard clinico--pathological parameters and CTC status………………………………… 6

Supplementary Table 4. Recurrence-free survival probability in patients with operable stage III-IV NSCLC according to standard clinico-pathological parameters and CTC status…………………………………………………………… 7

Supplementary Figures ………………………………………………………………………… **8**

Supplementary Figure 1. Naked nuclei detected in the blood of lung cancer-free individuals. ……………………………………………………….. 8

Supplementary Figure 2. Naked nuclei detected in the blood of non-small cell lung cancer patients ………………………………………………….. 9

Supplementary Table S1. Single circulating tumor cell (sCTC) prevalence and non-small cell lung cancer (NSCLC) patients' characteristics

	N (%)	N sCTC≥1 (%)	N sCTC≥2 (%)	N sCTC≥3 (%)	N sCTC≥5 (%)
All cases	74 (100)	34 (45.9)	15 (20.3)	9 (12.2)	4 (5.4)
**Sex**					
Female	33 (44.6)	13 (39.4)	2 (6.1)	2 (6.1)	1 (3.0)
Male	41 (55.4)	21 (51.2)	13 (31.7)	7 (17.1)	3 (7.3)
p-value		*.3542*	*.0081*	*.2831*	*.6237*
**Smoking habits**					
Current smoker	25 (33.8)	12 (48.0)	7 (28.0)	4 (16.0)	1 (4.0)
Former smoker	32 (43.2)	16 (50.0)	8 (25.0)	5 (15.6)	3 (9.4)
Never-smoker	16 (21.6)	6 (37.5)	0	0	0
Missing	1 (1.4)	0	0	0	0
p-value[a]		*.5719*	*.0308*	*.1915*	*.5699*
**Tumor stage**					
IA	27 (36.5)	12 (44.4)	4 (14.8)	3 (11.1)	1 (3.7)
IB	5 (6.8)	1 (20.0)	0	0	0
IIA	4 (5.4)	4 (100)	2 (50.0)	2 (50.0)	0
IIB	16 (21.6)	7 (43.8)	3 (18.8)	1 (6.3)	0
IIIA	11 (14.9)	6 (54.5)	4 (36.4)	1 (9.1)	1 (9.1)
IIIB	6 (8.1)	2 (33.3)	1 (16.7)	1 (16.7)	1 (16.7)
IIIC	0	0	0	0	0
IVA	5 (6.8)	2 (40.0)	1 (20.0)	1 (20.0)	1 (20.0)
p-value[b]		*>.9999*	*.3551*	*>.9999*	*.0760*
**Histological subtype**					
Adenocarcinoma	55 (74.3)	25 (45.5)	10 (18.2)	5 (9.1)	3 (5.5)
Squamous cell carcinoma	16 (21.6)	8 (50.0)	5 (31.3)	4 (25.0)	1 (6.3)
Other	2 (2.7)	1 (50.0)	0	0	0
Missing	1 (1.4)	0	0	0	0
p-value[c]		*.7825*	*.3030*	*.1081*	*>.9999*
**Grading**					
G1	4 (5.4)	2 (50.0)	0	0	0
G2	44 (59.5)	17 (38.6)	7 (15.9)	4 (9.1)	2 (4.5)
G3	24 (32.4)	14 (58.3)	8 (33.3)	5 (20.8)	2 (8.3)
G4	0	0	0	0	0
Missing	2 (2.7)	1 (50.0)	0	0	0
p-value[d]		*.1435*	*.1212*	*.1492*	*.5965*

[a] current/former versus never-smokers
[b] stage I/II versus III/IV
[c] adenocarcinoma versus squamous cell carcinoma
[d] G1/G2 versus G3/G4

Supplementary Table S1. Single circulating tumor cell (sCTC) prevalence and non-small cell lung cancer (NSCLC) patients' characteristics *(continued)*

	N (%)	N sCTC≥1 (%)	N sCTC≥2 (%)	N sCTC≥3 (%)	N sCTC≥5 (%)
**Visceral pleura invasion**					
PL0	56 (75.7)	26 (46.4)	13 (23.2)	8 (14.3)	3 (5.4)
PL1	8 (10.8)	5 (62.5)	1 (12.5)	1 (12.5)	1 (12.5)
PL2	5 (6.8)	1 (20.0)	0	0	0
PL3	2 (2.7)	1 (50.0)	1 (50.0)	0	0
Missing	3 (4.1)	1 (33.3)	0	0	0
p-value[e]		*>.9999*	*>.5001*	*.6730*	*>.9999*
**Peritumoral neoplastic angioinvasion**					
Absent	66 (89.2)	32 (48.5)	14 (21.2)	9 (13.6)	4 (6.1)
Present	6 (8.1)	1 (16.7)	1 (16.7)	0	0
Missing	2 (2.7)	1 (50.0)	0	0	0
p-value		*.2088*	*>.9999*	*>.9999*	*>.9999*
**Lymph-node status**					
Negative	48 (64.9)	22 (45.8)	10 (20.8)	6 (12.5)	2 (4.2)
Positive	25 (33.8)	11 (44.0)	5 (20.0)	3 (12.0)	2 (8.0)
Missing	1 (1.4)	0	0	0	0
p-value		*>.9999*	*>.9999*	*>.9999*	*.6027*

[e] PL0 vs PL1/PL2/PL3
Abbreviations: hetCLU, heterotypic cluster; homCLU, homotypic CTC cluster

Supplementary Table S2. Circulating tumor cell cluster (cCTC) status and NSCLC patients' characteristics

	N (%)	N cCTC+ve (%)	N hetCLU+ve (%)	N homCLU+ve (%)
All cases	74 (100)	24 (32.4)	23 (31.1)	2 (2.7)
**Sex**				
Female	33 (44.6)	10 (30.3)	10 (30.3)	0
Male	41 (55.4)	14 (34.1)	13 (31.7)	2 (4.9)
p-value		*.8055*	*>.9999*	*.4991*
**Smoking habits**				
Current smoker	25 (33.8)	12 (48.0)	11 (44.0)	1 (4.0)
Former smoker	32 (43.2)	8 (25.0)	8 (25.0)	1 (3.1)
Never-smoker	16 (21.6)	4 (25.0)	4 (25.0)	0
Missing	1 (1.4)	0	0	0
p-value		*.5553*	*.7616*	*>.9999*
**Tumor stage**				
IA	27 (36.5)	9 (33.3)	9 (33.3)	0
IB	5 (6.8)	2 (40.0)	2 (40.0)	0
IIA	4 (5.4)	1 (25.0)	1 (25.0)	0
IIB	16 (21.6)	6 (37.5)	5 (31.3)	2 (12.5)
IIIA	11 (14.9)	2 (18.2)	2 (18.2)	0
IIIB	6 (8.1)	1 (16.7)	1 (16.7)	0
IIIC	0	0	0	0
IVA	5 (6.8)	3 (60.0)	3 (60.0)	0
p-value		*.5974*	*.7858*	*>.9999*
**Histology**				
Adenocarcinoma	55 (74.3)	18 (32.7)	18 (32.7)	0
Squamous cell carcinoma	16(21.6)	5 (31.3)	4 (25.0)	2 (12.5)
Other	2 (2.7)	1 (50.0)	1 (50.0)	0
Missing	1 (1.4)	0	0	0
p-value		*>.9999*	*.7603*	*.0483*
**Grading**				
G1	4 (5.4)	2 (50.0)	2 (50.0)	0
G2	44 (59.5)	14 (31.8)	14 (31.8)	1 (2.3)
G3	24 (32.4)	8 (33.3)	7 (29.2)	1 (4.2)
G4	0	0	0	0
Missing	2 (2.7)	0	0	0
p-value		*>.9999*	*.7935*	*>.9999*

[a] current/former versus never-smokers
[b] stage I/II versus III/IV
[c] adenocarcinoma versus squamous cell carcinoma
[d] G1/G2 versus G3/G4
Abbreviations: hetCLU, heterotypic cluster; homCLU, homotypic CTC cluster

Supplementary Table S2. Circulating tumor cell cluster (cCTC) status and NSCLC patients' characteristics *(continued)*

	N (%)	N cCTC+ve (%)	N hetCLU+ve (%)	N homCLU+ve (%)
**Visceral pleura invasion**				
PL0	56 (75.7)	16 (28.6)	15 (26.8)	2 (3.6)
PL1	8 (10.8)	2 (25.0)	2 (25.0)	0
PL2	5 (6.8)	3 (60.0)	3 (60.0)	0
PL3	2 (2.7)	2 (100)	2 (100)	0
Missing	3 (4.1)	1 (33.3)	1 (33.3)	0
p-value		*.2007*	*.2075*	*>.9999*
**Peritumoral neoplastic angioinvasion**				
Absent	66 (89.2)	24 (36.4)	23 (34.8)	2 (3.0)
Present	6 (8.1)	0	0	0
Missing	2 (2.7)	0	0	0
p-value		*.1692*	*.1674*	*>.9999*
**Lymph-node status**				
Negative	48 (64.9)	17 (35.4)	16 (33.3)	1 (2.1)
Positive	25 (33.8)	7 (28.0)	7 (28.0)	1 (4.0)
Missing	1 (1.4)	0	0	0
p-value		*.6055*	*.7920*	*>.9999*

[e] PL0 vs PL1/PL2/PL3
Abbreviations: hetCLU, heterotypic cluster; homCLU, homotypic CTC cluster

Supplementary Table S3. Recurrence-free survival probability in patients with operable stage I-II NSCLC according to standard clinico-pathological parameters and CTC status

Variable	N cases	N recurrence events	Survival proportion (%)	HR 95% (CI)	p-value
**pStage**					
**I**	30	7	70		
**II**	14	6	51	2.19 (0.65-7.36)	*.1452*
**Grading**					
**G1/G2**	33	9	70		
**G3/G4**	11	4	51	1.52 (0.41-5.57)	*.4820*
**Lymph-node status**					
**Negative**	38	11	65		
**Positive**	6	2	56	1.47 (0.26-8.36)	*.1092*
**CTC status**					
**Negative**	18	3	82		
**Positive**	26	10	55	2.27 (0.75-6.86)	*.2003*
**sCTC count**					
**<2**	36	7	77		
**≥2**	8	6	15	5.15 (1.10-24.33)	*.0009*
**sCTC count**					
**<3**	39	10	69		
**≥3**	5	3	27	3.99 (0.47-33.57)	*.0216*
**hetCLU status**					
**Negative**	29	7	73		
**Positive**	15	6	55	1.42 (0.46-4.35)	*.5267*

Abbreviations. "HR", hazard ratio; "CI", confidence interval; "CTC", circulating tumor cell(s); "hetCLU", heterotypic circulating tumor cell clusters.

Supplementary Table S4. Recurrence-free survival probability in patients with operable stage III-IV NSCLC according to standard clinico-pathological parameters and CTC status

Variable		N cases	N recurrence events	Survival proportion (%)	HR 95% (CI)	p-value
**pStage**						
	**III**	13	8	33		
	**IV**	5	5	0	2.78 (0.68-11.33)	*.0537*
**Grading**						
	**G1/G2**	8	5	38		
	**G3/G4**	8	6	18	1.33 (0.41-4.35)	*.6261*
**CTC status**						
	**Negative**	6	3	44		
	**Positive**	12	10	11	3.12 (1.05-9.27)	*.0573*
**sCTC count**						
	**<2**	12	9	21		
	**≥2**	6	4	25	1.10 (0.32-3.49)	*.9223*
**sCTC count**						
	**<3**	15	11	19		
	**≥3**	3	2	33	0.73 (0.19-2.82)	*.6693*
**hetCLU status**						
	**Negative**	13	8	31		
	**Positive**	5	5	0	3.44 (0.76-15.50)	*.0129*

Abbreviations. "HR", hazard ratio; "CI", confidence interval; "CTC", circulating tumor cell(s); "hetCLU", heterotypic circulating tumor cell clusters.

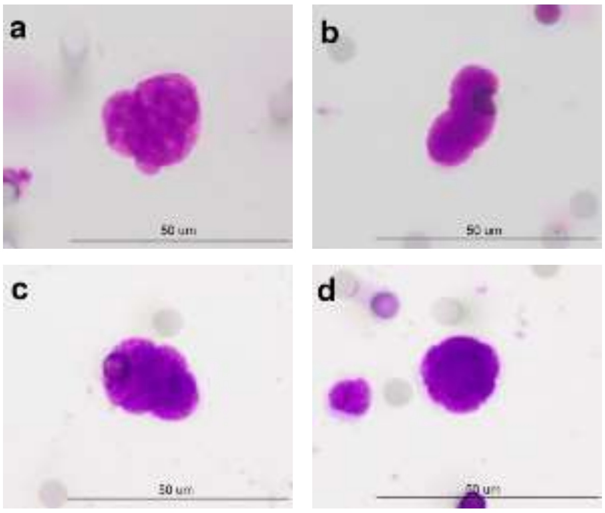

Supplementary Figure S1. Naked nuclei detected in the blood of lung cancer-free individuals. Images depict naked nuclei, i.e. without apparent cytoplasm, with the longer diameter >20 μm and irregular border, sometimes (**a**, **c**) overlapped, (**b**) oblong or (**b**, **d**) hyperchromatic, on porous membranes stained with May-Grünwald Giemsa. Objective magnification 40x.

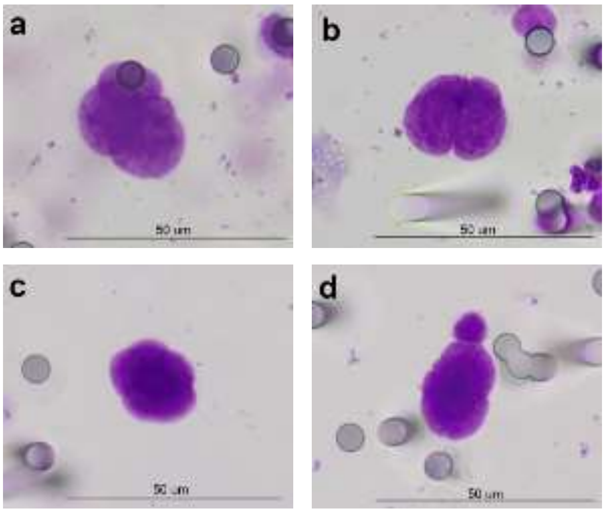

Supplementary Figure S2. Naked nuclei detected in the blood of non-small cell lung cancer patients. Images depict naked nuclei, i.e. without apparent cytoplasm, with the longer diameter >20 μm and irregular border, sometimes (**a**) overlapped, (**b**) juxtaposed, (**b**-**d**) hyperchromatic, and (**d**) clustered with other smaller nuclei consistent with possible leukocytes, on porous membranes stained with May-Grünwald Giemsa. Objective magnification 40x.
